# Application of Af4-Multidetection to Liraglutide in Its Formulation: Preserving and Representing Native Aggregation

**DOI:** 10.3390/molecules27175485

**Published:** 2022-08-26

**Authors:** Valentina Marassi, Marco Macis, Stefano Giordani, Lucia Ferrazzano, Alessandra Tolomelli, Barbara Roda, Andrea Zattoni, Antonio Ricci, Pierluigi Reschiglian, Walter Cabri

**Affiliations:** 1 Department of Chemistry G. Ciamician, University of Bologna, 40126 Bologna, Italy; 2 byFlow srl, 40129 Bologna, Italy; 3 Fresenius Kabi iPSUM, Via San Leonardo 26, 45010 Villadose, Italy

**Keywords:** FFF-multidetection, flow-field flow fractionation, native aggregation state, sameness, therapeutic peptides, Liraglutide, active pharmaceutical ingredient (API), finished dosage form (FDF), reference listed drug, RLD

## Abstract

Aggregation is among the most critical parameters affecting the pharmacological and safety profile of peptide Active Pharmaceutical Ingredients (APIs). For this reason, it is of utmost importance to define the exact aggregation state of peptide drugs, particularly when the API is marketed as a ready-to-use solution. Consequently, appropriate non-destructive techniques able to replicate the peptide environment must be employed. In our work, we exploited Asymmetrical Flow Field-Flow Fractionation (AF4), connected to UV, dRI, fluorescence, and MALS detectors, to fully characterize the aggregation state of Liraglutide, a peptide API used for the treatment of diabetes type 2 and chronic obesity. In previous studies, Liraglutide was hypothesized to assemble into hexa-octamers in phosphate buffer, but no information on its behavior in the formulation medium was provided up to now. The method used allowed researchers to work using formulation as the mobile phase with excellent recoveries and LoQ/LoD, discerning between stable and degraded samples, and detecting, when present, aggregates up to 10^8^ Da. The native state of Liraglutide was assessed and found to be an association into pentamers, with a non-spherical conformation. Combined to benchmark analyses, the sameness study was complete and descriptive, also giving insight on the aggregation process and covalent/non-covalent aggregate types.

## 1. Introduction

Therapeutic peptides represent a huge area of interest in the pharmaceutical industry. So far, more than 50 candidates have been approved as therapeutics and many others are undergoing clinical trials [1,2,3]. Compared to small molecule drugs, such as higher specificity, low toxicity, and high tolerance by the human body [4]. Moreover, advances in manufacturing strategies, primary structure modifications, and computational strategies in formulation development facilitate investment in the development of these kind of drugs [5,6].

Peptide candidates can be produced either with chemical or recombinant strategies (upstream process), according to the length of the sequence and from the presence of non-proteinogenic amino acids in the primary structure [7,8]. In both cases, the crude peptides obtained require a purification step (downstream process) to achieve the quality target requested by the national regulatory authorities, using ion exchange and size exclusion chromatography, precipitation, and extraction, even though the reference technique remains ion pair reversed-phase liquid chromatography [9,10]. Once the peptide Active Pharmaceutical Ingredient (API) is produced and purified, and usually lyophilized, it undergoes formulation to reach its Finished Dosage Form (FDF). This phase comprises a series of studies: pre-formulation (evaluation of physicochemical properties before and after combination to excipients), analytical profiling (structural characterization and impurity determination), and evaluation of pharmaceutical properties [11,12]. Commercialization of protein drug candidates has to further face many road barriers: changes in external and/or internal variables, including temperature, pH, and chemical environment, may lead to processes such as denaturation, aggregation, or precipitation, causing destabilization of the protein structure and affecting its functioning [13].

Among all these physical and chemical deterioration pathways, peptide aggregation, which is known to potentially trigger immunogenicity in patients, represents one of the most critical attributes of the peptide FDFs. This undesired phenomenon is usually fought during drug development by adopting suitable and appropriate formulation buffers or by modifying the native peptide primary sequence [14]. Further evidence of the importance of this aspect is the request set by the Food and Drug Administration (FDA) for the aggregation properties of the generic version of already commercialized peptides [15], that must show “sameness” with the product already present in the market (Reference Listed Drug, RLD). Even though this 2021 FDA guidance refers only to a small number of generic peptides that are manufactured by alternative methods (synthetic vs. recombinant), this guidance is now considered a standard approach for the evaluation of all generic commercialized peptides.

There are many techniques for monitoring peptide aggregation, widely used also for protein aggregation evaluation; in fact, though peptides are considerably smaller than proteins, they can also aggregate into high MW species which can be detected with the same approach. Traditional methods are, for example, microscopy and turbidimetry. However, the former is time-heavy and requires sample drying while turbidimetric results cannot give precise information on the type of aggregate formed [16]. Historically, determination of aggregation in peptide pharmaceuticals has relied upon size-exclusion chromatography (SEC). While SEC can be an effective tool in terms of sensitivity, precision, and throughput, there are some significant technical limitations of SEC that can lead to erroneous estimates of aggregate levels in peptide products. SEC is, in fact, characterized by a limited size resolution range which does not exceed 10^7^ Da (corresponding to an upper size limit near 100 nm) [17]. The results can also be misleading due to the limitations imposed on mobile phase composition, which has to be compatible with the stationary phase of the column (analysis not in native conditions). Moreover, it is possible to observe adsorption and/or dissociation processes caused by the interaction of the sample with the stationary phase [18]. For this reason, regulatory bodies, such as the FDA and the EMA (European Medicinal Agency), currently require the use of techniques orthogonal to SEC, such as Sedimentation Velocity Analytical Ultracentrifugation (SV-AUC) and Asymmetrical Flow Field-Flow Fractionation (AF4), to verify SEC data.

While SV-AUC is limited by comparable size range to SEC, the AF4 methods can cover a measurement range from 1 nm up to 1 μm [19]. AF4 also works with an empty channel which prevents adsorption and dissociation problems due to the stationary phase. This makes this technique also very flexible since mobile phase can be tuned on samples requirements and it is often possible to work in native conditions [19,20,21,22].

AF4, along with the other Field Flow Fractionation (FFF) techniques, is exploited to analyze different biological matrices, such as serum proteins [23] and antibodies [24,25], to simplify serum [26] and isolate lipoproteins, also in its miniaturized variants. These techniques are also used in the analysis of DNA and in the QC steps of various pharmaceutical compounds [25,27]. The importance of AF4 systems coupled with dRI and MALS detectors (AF4-multidetection) to properly detect high molecular weight species is remarked upon in several scientific works. AF4-multidetection was used for size characterization of drug-delivery systems and other supramolecular systems involving protein-mediated aggregation [19,28,29,30]. Last, aggregation studies of proteins and mAbs comparing AF4 and SEC [31] also remarked the value of AF4 in correctly detecting bigger aggregates and work in more flexible conditions. 

Within this framework, we focused our attention on Liraglutide, a peptide API used in the treatment of diabetes Type 2 and chronic obesity with the brand names Victoza and Saxenda, respectively [32,33]. Liraglutide FDF is available as an injectable solution composed by the API at a concentration of 6 mg/mL, phenol, propylene glycol, and sodium phosphate to adjust pH to about 8.15. Furthermore, Liraglutide is one of the peptides listed in the FDA 2021 guidance; for this reason, any generic candidate needs a deep characterization of the aggregation properties and an extensive comparison with the RLD to guarantee “sameness” and safely access the market [15]. However, although the pharmacological properties of Liraglutide have been established, there is a lack of knowledge concerning the aggregation tendency of Liraglutide, even for the product currently present in the market (RLD). In fact, only few works are currently present in the literature, mainly focused on how the pH of the environment influences the aggregation of the peptide and its kinetics [34,35,36]. Steensgaard et al. [37] showed that Liraglutide oligomerizes in a concentration-independent manner forming, predominately, heptamers in the concentration range 0.004–4.501 mg/mL. Insights were also given by circular dichroism [38]. Frederiksen et al. [39] instead performed molecular dynamic simulation studies to estimate the most stable aggregation levels of Liraglutide and compared the results with the ones obtained through Small-Angle X-Ray Scattering (SAXS) and FFF-UV-DAD analysis. These measurements, performed on the Liraglutide commercialized product (brand name: Victoza) and using PBS as mobile phase, described a hexameric structure for the aggregates. On the other side, the theoretical studies estimated hexa-, hepta-, and octameric structures as the most likely ones. All these studies lack several key aspects concerning the Liraglutide analytical problem: (a) none of them is performed with the API dissolved in the diluent present in the Victoza/Saxenda FDF (aqueous solution at pH around 8.15 with phenol and propylene glycol); (b) most Liraglutide samples are not commercial products but are derived by a purified standard; and (c) none of the studies worked at the concentration of the FDF (6 mg/mL) but on more diluted systems. Thus, these kinds of results, aside from being a helpful stepping-stone in this field, have no practical use for the characterization of the already existing products and for the establishment of a general analytical tool for the evaluation of the aggregation properties between peptide samples.

For these reasons, in this work we performed measurements on several Liraglutide samples in formulation with a double aim: (1) performing a sameness study while preserving the native state of the samples in the diluent used for the FDF; and (2) evaluate the aggregation state of the peptide in solution before the lyophilization step and after the formulation step. To achieve our goals, we exploited an AF4 platform coupled to UV, fluorescence, refractive index (dRI), and multi-angle light scattering detection, which showed excellent recovery and LOD and allowed a thorough characterization (mass- and size-wise), also overcoming technical limitations caused by working with formulation as a mobile phase. A set of samples, including pre-lyophilization API in solution, a FDF produced internally, and two RLD, namely Victoza and Saxenda, were selected and characterized in their native state. The results from FDF and RLD were evaluated also to the light of SEC-MALS analysis to obtain a complete scenario regarding Liraglutide in suspension. This approach is able to answer to FDA recommendations and is, auspicably, a starting point for further studies of drug peptides aggregation, to ensure representative results and product safety.

## 2. Results and Discussion

### 2.1. Method Development and Optimization of Detection Parameters

**Figure 1:**
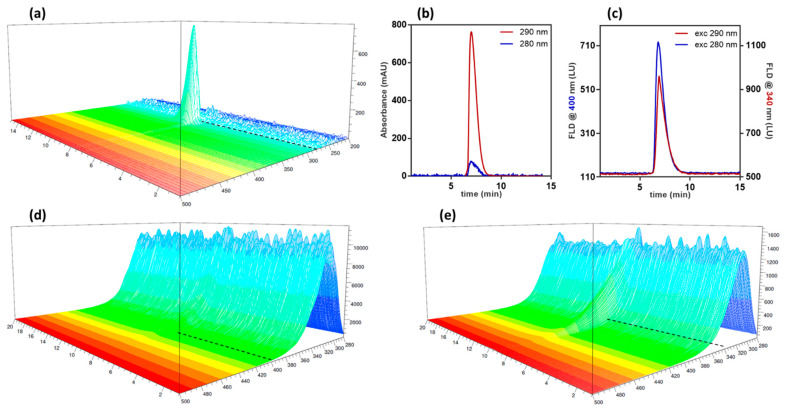
(**a**) Absorption spectrum of Liraglutide in formulation buffer analysed in Focus-FIA. Dashed line: absorption at 280 nm. (**b**) Overlay of UV absorption at 280 (blue) and 290 (red) nm. (**c**) Comparison between fluorescence signals at optimal wavelength upon excitation at 280 nm (blue) or 290 nm (red). (**d**,**e**) Emission spectrum of Liraglutide upon excitation at 280 nm (**d**) and 290 nm (**d**,**e**). Dashed line: emission signal at 340 nm.

The AF4-multidetection method developed in this work used Liraglutide formulation as the mobile phase. If, on one side, this approach is able to correctly represent the aggregation state of the peptide in suspension, on the other, it is more difficult to find optimal separation and detection conditions, since the mobile phase is not optimized towards the instrumental setup. Therefore, the use of a set of different, uncorrelated detectors is recommended to overcome the limitations of the single techniques, such as signal interferences from the absorbents in the mobile phase and baseline drift due to eluent viscosity. In the case of Liraglutide formulation (see materials and methods), the presence of phenol in the mobile phase caused absorption and emission in the same wavelength ranges of the conventional values used for proteins and peptides (280 nm absorption and excitation, 340 nm emission). The presence of ethylene glycol increased mobile phase viscosity, inferring baseline drifts to the concentration detectors (UV, dRI) during the separation methods, which are subject to flow and pressure changes. For this reason, absorption at 280 nm could not be exploited because of the high noise/signal ratio and the lack of a relative maximum, and the 290 nm wavelength was preferred. Contrarily to UV and dRI, fluorescence is unaffected by pressure and flow changes and can be a reliable source, also for detection of different populations. Excitation at 280 and at 290 nm were, thus, explored to gain a reliable signal for recovery study. The UV absorption spectrum of Liraglutide in formulation buffer and the emission spectra upon excitation at 280 and 290 nm are shown in Figure 1.

Absorption below 280 nm (Figure 1a) is completely covered by interferences, whereas signal at 290 nm (red trace, Figure 1b, compared to absorption at 280 nm, blue trace) is intense and baseline noise is low. With this information in hand, we proceeded to scan for emission upon excitation at these two wavelengths, obtaining the relative emission spectra (Figure 1d,e). Baseline noise was found acceptable at 400 nm emission (Figure 1d, dashed line) and after 340 nm (Figure 1e, dashed line), i.e., where the noise/signal ratio was the lowest in the two cases. The corresponding emission profiles are shown in Figure 1c. Interestingly, though absorption at 280 nm is not performing, its emission showed the lowest LOD and LOQ observed, which resulted to be 2.1 nmol and 7 nmol, respectively.

**Figure 2:**
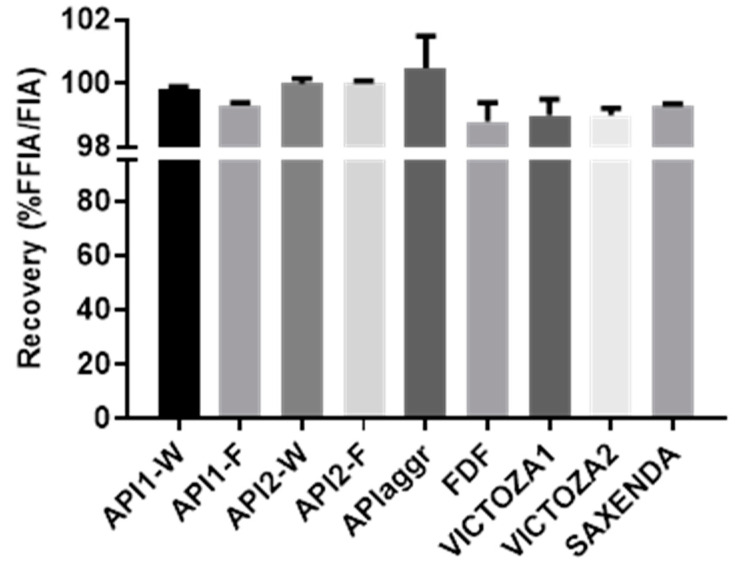
Recovery calculated for all Liraglutide FDF, expressed as area % between a Focus-FIA and a FIA.

The following steps of method development involved the selection of the channel membrane and recovery calculation: for this purpose, a Polyethersulfone (PES) membrane with a molecular weight cutoff (MWCO) of 1 kDa was chosen, since other materials and variants could not ensure a cutoff below the peptide molecular weight. Recovery, calculated in triplicate, was evaluated both for BSA as standard protein and for all Liraglutide samples (API, FDF, RLD, and aggregated API; see Table 1). BSA showed quantitative recovery (>99.9%), whereas recovery for Liraglutide, while optimal, ranged between 98 and 100%. As shown in Figure 2, higher recovery was observed for the API samples (aggregated or untreated) while a slightly lower value was found for FDF and commercial products (between 98 and 99%), probably due to the different aging time between the API samples (freshly suspended in the formulation media at the time of the experiment) and the FDF and RLD ones (already suspended in the formulation buffer at the beginning of the product shelf life).

**Figure 3:**
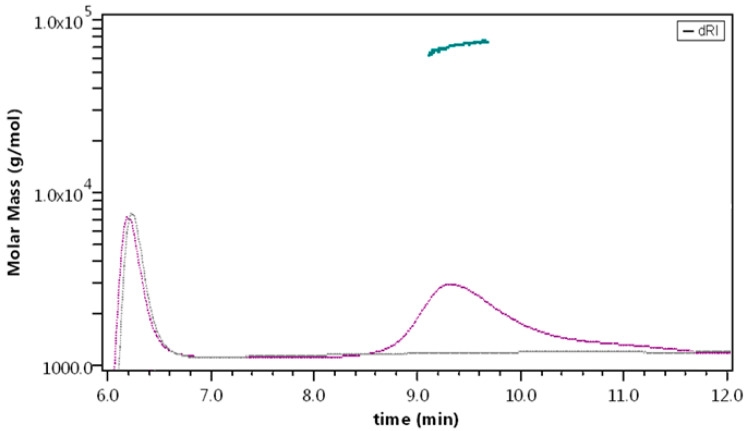
dRI signal overlay of a blank (grey) and BSA (pink) injection with the optimized method. Green distribution: molar mass calculation of BSA monomer.

A focus flowrate of 1.50 mL/min (inject flow: 0.2 mL/min) and a longitudinal flowrate of 0.50 mL/min were selected; different focus flowrates and focusing times did not influence recovery but worsened the baseline drift. The crossflow velocity was then scanned in the 1.0–2.5 mL/min range using API1-W as a test sample: only the highest crossflow rate applied resulted in a good separation from the void peak, while higher flowrates could not be employed due to pressure limits of the system. To shorten analysis time, and allow possible highly-aggregated species to be eluted before the field is released, the method was further modified to a gradient decreasing crossflow rate to zero in 20 min. After six minutes from the start of the run, when the flow control switches from focus to elution, a system peak appears in all analyses (blank included), due to the change in system pressure. Applying the crossflow also showed the baseline drifts above-mentioned, both for the dRI and UV detector, while fluorescence remained unaffected in all cases. The applicability of the developed setup was also confirmed by injections of BSA, whose mass was correctly calculated using dRI as a concentration source (Figure 3).

The developed method adhered to the standards a validated method must satisfy according to the harmonized guideline ICHQ2R1 and ISO/TS 21362 [40,41] and was, therefore, considered a standardized method and used to characterize all samples. 

### 2.2. Characterization of API and Aggregated API

**Figure 4:**
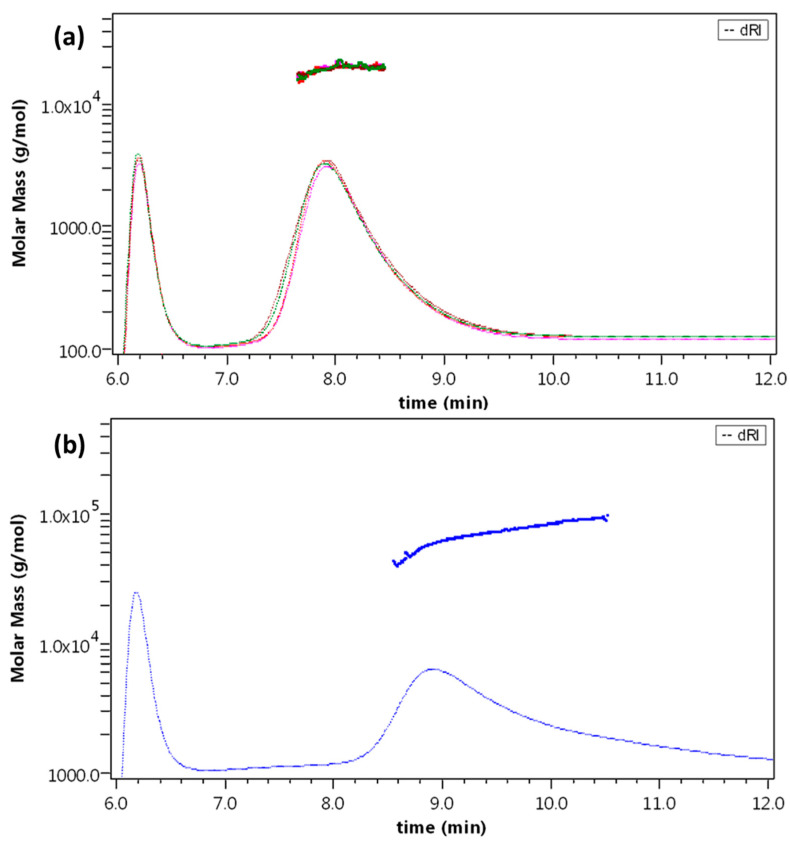
(**a**) dRI fractogram and molar mass overlay of API1-W (red), API1-F (purple), API2-W (green), and API2-F (violet), representative runs. (**b**) dRI fractogram and molar mass overlay of APIaggr, representative run.

The API samples were characterized with the AF4-multidetection method, both after resuspension in water and in formulation at the concentration of 6 mg/mL. In both cases, Liraglutide was eluted with a single peak at 7.8 min, with API1 and API2 resulting overlapped and identical in profile and molar mass distribution (Figure 4a). 

The molar mass obtained from light scattering was found between 19 and 20 kDa for all samples (API1-W: 19.6 ±0.4; API1-F: 19.8 ± 0.2; API2-W: 19.2 ± 0.8; and API2-F: 19.7 ± 0.5 kDa), whereas no evidence of monomer was observed also from the fluorescence profile. The *v* value calculated (as obtained from a conformation plot, see Section 3) averaged 0.78, indicating a non-spherical, non-compact conformation [42].

The capability of the method to discern different forms of Liraglutide was monitored by the analysis of a sample of thermally stressed, aggregated API: in this case, no species are observed before 9 min elution time, where a band is eluted with a broad molar mass distribution averaging 73.7 kDa and corresponding to aggregates of >20 units (Figure 4b). The found *v* value was negative, meaning that a mass increase corresponds to a size decrease [43] and the structure is extremely compact, possibly due to irreversible denaturation and covalent aggregation. In this case, a second population is detected by fluorescence (9 ± 1% of fluorescence area) at 26 min, corresponding to the field release (i.e., zero crossflow), with an estimated molar mass up to 10^8^ Da.

### 2.3. Characterization of FDF and RLD Samples

**Figure 5:**
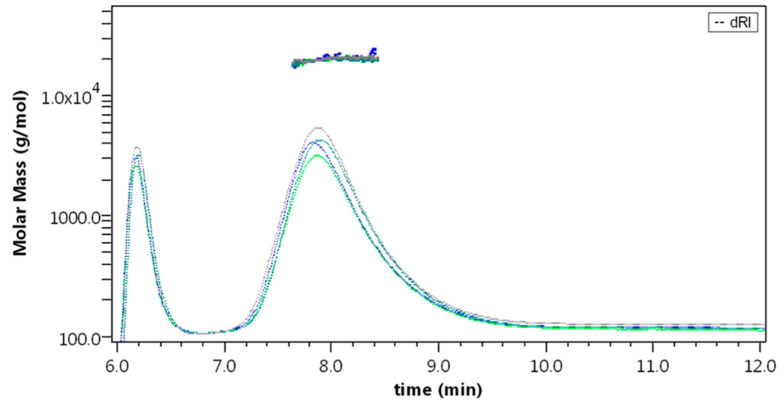
dRI fractogram and molar mass overlay of FDF (blue), Victoza1 (green), Victoza2 (teal), and Saxenda (blue), representative runs.

The FDF and the RLD (Victoza, Saxenda) samples were also submitted to the same method. The FDF sample appeared as a single band, with the same retention time (7.8 min) and molar mass distribution (19.9 ± 0.4 kDa) as previous samples; likewise, both the Victoza lots and the Saxenda lot analysed contained a single population corresponding to Liraglutide oligomers (Victoza1: 19.2 ± 0.7 kDa; Victoza2 20.1 ± 0.4 kDa; and Saxenda: 19.9 ± 0.3 kDa). All samples are overlaid in Figure 5.

Like previous samples, the calculated *v* values corresponded to 0.7, indicating a branched/extended conformation; no trace of monomer or higher MW oligomers was detected.

### 2.4. Mass Comparison and Sameness Evaluation

**Figure 6:**
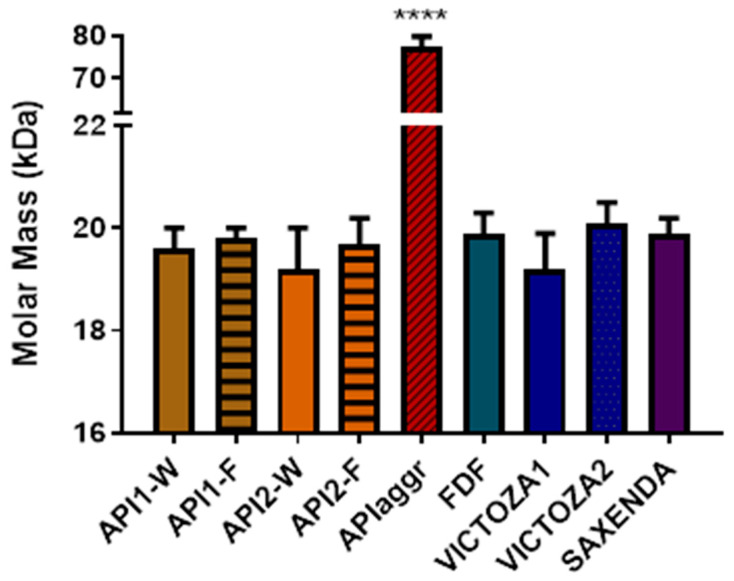
Molar mass distributions calculated for all samples. ****: *p* < 0.0001.

All samples are grouped together and showed as histograms representing their molar mass in Figure 6. Statistical evaluations (performed through one-way ANOVA) confirmed that there is no significant difference between all samples, apart from the aggregated API (*p* < 0.0001 against all other samples), confirming the identity between commercial and R&D products.

Interestingly, sameness was also assessed for freshly dissolved APIs (API1-W, and API2-W), suggesting that the aggregation of Liraglutide in formulation is a fast process which yields a single aggregate type, which then stabilizes the structure and prevents further aggregation. This information was further verified by lowering the focusing time and confirming that no difference in the molar mass distribution was detected.

The associated form found for Liraglutide in formulation is numerically equal to 5.2 units, indicating the main presence of a pentamer. When the sample is subjected to heating (APIaggr), the thermal stress imposed to the sample generate a stable aggregation, likely through unfolding, leading to a very compact denaturated aggregates averaging 20 units.

### 2.5. HPLC Analyses

The aggregation state of Liraglutide, together with the sameness evaluation of the finished product and the commercial products, was also conducted through HPLC-MALS as benchmark technique. In this case, due to the limitations in the stationary phase composition, the analyses were conducted in a mobile phase containing 30% acetonitrile.

**Figure 7:**
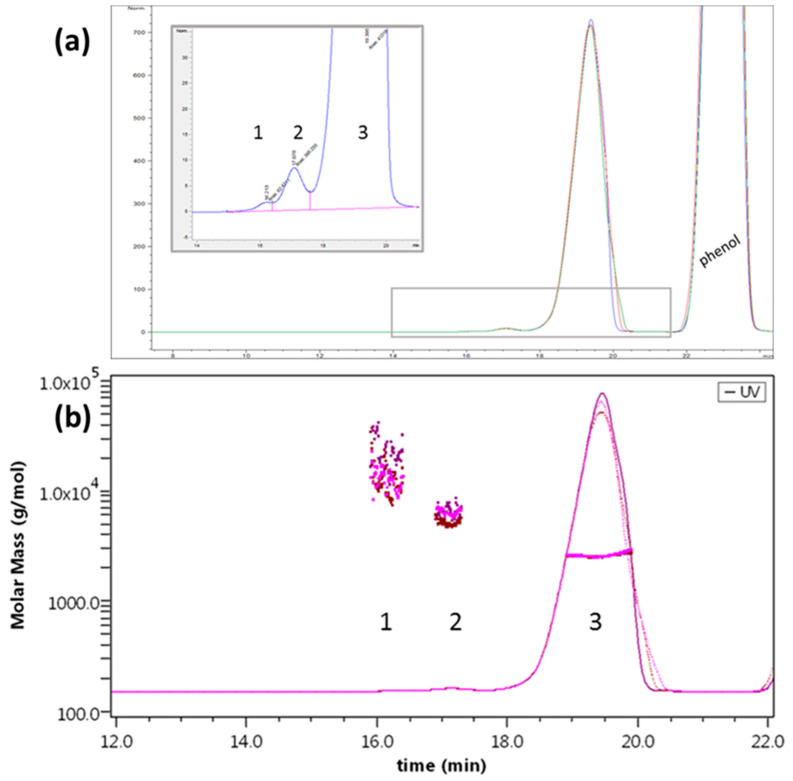
SEC analysis of Liraglutide. (**a**) Separation profile. Inset: zoom on the higher MW population (dimer, tetramer). (**b**) Molar mass calculation obtained from MALS. Overlay of three replicates of a representative sample.

The results present a main peak at 19 min for all the samples tested, followed by two minor peaks at 16 and 17 min, respectively. At 23 min the peak corresponding to phenol (contained in the formulation medium) is eluted (Figure 7a).

**Figure 8:**
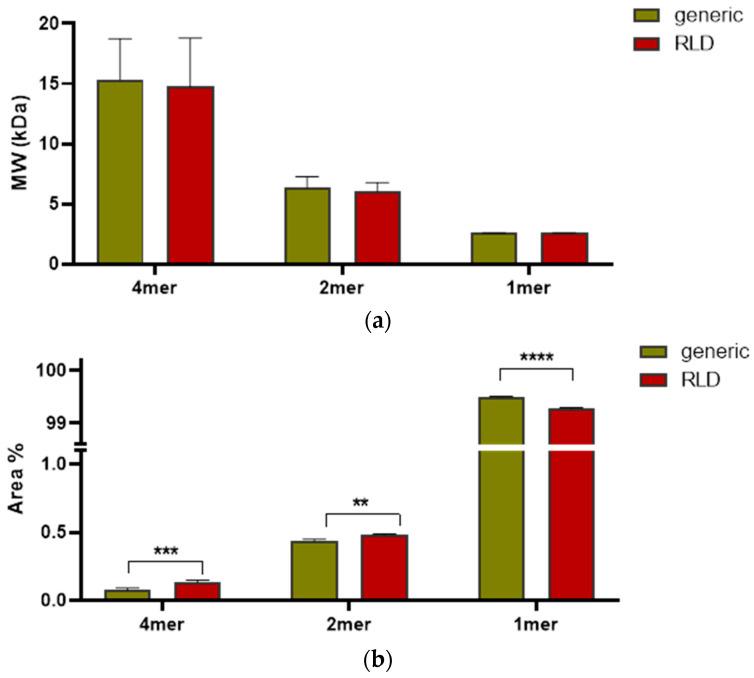
Comparison between different Liraglutide samples. (**a**) Measured molar mass; (**b**) relative abundance of aggregated species. ****: *p* < 0.0001. ***: *p* < 0.001. **: *p*< 0.01 (two-way ANOVA).

Mass calculation (Figure 7b) results in a slight under-estimation of the monomer weight, an effect (that of MW deviation) observed with the use of a mixed solvent due to preferential solvation [44]. The species detected correspond to monomer, dimer, and tetramer (*n* = 3), and the two preparations show the same peaks, retention time, and molar mass distributions. In terms of relative abundance, the amount of monomer is above 99.5% for both preparations (Figure 8a,b), though some differences could be detected between samples. We observed that for lots closer to the expiration date, the percentage of monomer measured was lower, suggesting that aging of the sample is linked to the amount of irreversible aggregation.

Interestingly, SEC analyses are able to revert the aggregation state found in FFF analyses and highlight the main presence of the monomer, confirming the non-covalent nature of the pentamer. Moreover, the size of the aggregates can give information on the aggregation pattern (e.g., dimer–dimer, dimer–monomer combining) or can indicate sample deterioration, which could follow the same process but be irreversible.

The combination of unprecedented FFF data in formulation to SEC analyses is crucial to satisfy FDA requirements for peptide drug commercialization. Indeed, ANDA applicants are encouraged to apply orthogonal analytical methods to characterize—among the rest—secondary structure and oligomer/aggregation and “demonstrate that the proposed synthetic peptide’s active ingredient is the ‘same as’ the active ingredient in the RLD” [15]. Because the afore-mentioned properties may be affected by the formulation of the drug product, an applicant should evaluate them carefully and in an environment as close as possible to the real one.

The use of AF4 and SEC represent a suitable approach for this kind of study since the first portrays the real aggregation state in native conditions, and the second removes labile aggregation. Together, they show that Liraglutide is not polymerized, but aggregated in stable non-covalent oligomers (pentamers) which are removed in SEC conditions. Furthermore, since the reversed elution order between the two techniques favors the use of FFF for High MW species, the 10^8^ Da species found for the aggregated API sample would go undetected in SEC analyses, thus lacking a critical information for the understanding of the aggregation properties of Liraglutide and posing an unknown health risk. At the same time, FFF is necessary also for low MW species for its ability to accurately representing the native state, where the monomer is not present. Moreover, FFF analyses can be performed in formulation buffer since there are very few limitations on choice of mobile phase, therefore adhering to the most recent requirements imposed by the FDA for the aggregation and molecular conformation comparative study between the peptide samples (sameness studies). Together, SEC and FFF represent a complete picture of the native aggregation state (FFF) and aggregate type (SEC) of Liraglutide.

## 3. Materials and Methods

### 3.1. Samples

Bovine Serum Albumin (BSA, Merck KGaA, Darmstadt, Germany); aggregated Liraglutide: AF00195I and AF00195L, 6 mg/mL; API: ALICEP 00136C and API MC 00291D, lyophilized and resuspended to 6 mg/mL in water or formulation; finished dosage form sample: FDF lot #33124-46, 6 mg/mL; RLD (Fresenius Kabi): Victoza Lot #HS65J01 and Lot #KS6AH74, 6mg/mL. RLD (Novo Nordisk): Saxenda Lot #JP54138, 6mg/mL. The composition of formulation buffer is as follows: disodium phosphate dihydrate, 0.476 g/L; propylene glycol, 0.47 g/L; phenol, 1.8 g/L; and water for injection. The lyophilized samples were resuspended to the same concentration of the FDF and RLD. The Liraglutide sample names, concentration, dilution media, and codename are listed in Table 1.

**Table 1:** Sample list.

	State	Concentration	Medium	Name	Injection Volume (µL)
API (Lot #ALICEP-001-36C)	lyophilized	6 mg/mL	Water, formulation	API1-w API1-f	30
API (Lot #MC-002-91D)	lyophilized	6 mg/mL	Water formulation	API2-w API2-f	30
Finished dosage form (FDF) (Lot #33124-46)	solution	6 mg/mL	Formulation	FDF	30
Victoza (Lot #HS65J01) (Lot #KS6AH74)	solution	6 mg/mL	Formulation	Victoza1 Victoza2	30
Saxenda (Lot #JP54138)	solution	6 mg/mL	Formulation	Saxenda	30
Stressed API (Lot #AF-001-95L)	solution	3 mg/mL	Formulation	APIaggr	60
Finished dosage form (FDF) (Lot #33387-18)	solution	6 mg/mL	Formulation		30
Saxenda (Lot #JP54138)	solution	6 mg/mL	Formulation		30

All chemicals were purchased from Merck KGaA, Darmstadt, Germany.

### 3.2. AF4-UV-FLD-RI-MALS Analysis

AF4-MALS was performed by using a 1100 Series HPLC system (Agilent Technologies, Palo Alto, CA, USA), connected to a module to control AF4 flow rates and operations (Eclipse 3, Wyatt Technology Europe, Dernbach, Germany). On-line detection of the eluted species was performed with an Agilent 1100 DAD UV/Vis spectrophotometer, an Agilent 1200 Fluorescence detector, a MALS DAWN HELEOS detector (Wyatt Technology Corporation, Santa Barbara, California), and an Optilab rEX refractive index detector (Wyatt Technology Corporation, Santa Barbara, California). Carrier solutions were degassed using an on-line vacuum degasser Agilent, 1100 series (Agilent Technologies).

The separation device is a flat channel (Wyatt Technology Europe) with a trapezoidal shape and capillary height. For analysis, sample solutions are injected in the channel and focused, allowing the sample to concentrate in a narrow band. When the elution starts, separation is gained by the combination of a longitudinal and a perpendicular hydrodynamic field. The channel was 152 mm long, equipped with a polyethersulfone (PES) membrane (Nadir), with 1 kDa molecular weight cutoff. The channel spacer was 350 μm thick, with trapezoidal shape (upstream width b0  =  16 mm; downstream width bL  =  4 mm).

The flow rate program was set up as follows: 1 min focus flow (1.5 mL/min) was applied to equilibrate the flows in the channel. Then 5 min injection in focus mode was applied in order to allow the sample to reach the channel. After the focus step, the elution starts with a gradient cross-flow step starting from 2.5 mL/min into 0 mL/min linearly in 20 min. Elution medium was formulation buffer.

Prior to size characterization, recovery was evaluated with a Flow-Injection Analysis (FIA) and a Focus-FIA. A FIA is a shortened, non-separative method: the sample is injected into the channel in absence of cross/focus flow and it reaches the detector without separation. It allows evaluating the signal related to 100% recovery of sample. A Focus-FIA is a FIA with an additional preliminary focusing step, where the sample is subject to the focus flow and narrowed in a thin band at the beginning of the channel [45,46]. In FIA, the entirety of the sample reaches the detector, while in Focus-FIA the sample components smaller than the membrane cutoff are filtered out, and only the colloidal portion of the sample goes through the detector. The ratio between the areas under signal curve obtained in Focus-FIA and FIA (%Focus-FIA/FIA) gives direct calculation of sample recovery [47]. Method precision was assessed both on retention times and on signal intensity by performing three independent replicates for BSA and Liraglutide. The same number of replicates (*n* = 3) were carried out for the analysis of each Liraglutide sample. The method reproducibility was verified in the method development step to ensure robustness. The limit of detection was calculated as the injected amount (nmol) at which the signal is three times the baseline standard deviation of the baseline, and the limit of quantification (LOQ) was calculated as the injected amount (nmol) at which the signal is 10 times the standard deviation of the baseline. Linearity was verified by Mandel’s fitting test within the concentration range explored in the performed analyses ± 50%. Multi-angle light scattering (MALS) was used to calculate the molar mass of eluted species, using Zimm model. It allows for the absolute determination of particle radius of gyration (Rg) by measuring the net intensity of light scattered by such particles at a range of fixed angles and, given the dn/dc and absorptivity values of the analysed molecules, the molar mass value of the eluted species [29]. The correlation between Rg and molar mass distributions provides information on the particle conformation in suspension through the calculation of a scaling exponent *v*, or *v* value. The *v* value is the slope in a double logarithmic logMW - logRg plot, and is theoretically defined for spheres as *v* = 0.33, random-coil *v* = 0.5–0.6, branched polymers = 0.7–0.8, and rod-like structures *v* 1 [48,49]. Data were processed by GraphPad Prism.

### 3.3. HPLC-UV-dRI-MALS

HPLC analyses were carried out with a Shodex HPLC Protein KW-802.5, 300 × 8.0 mm, 5 μm. The mobile phase used was 1 g/L L-Arginine: Acetonitrile: acetic acid, 35:50:15 (isocratic, 0.5 mL/min). Molar mass was calculated using dRI as the concentration source. The samples used were Liraglutide FDF Lot #33387-18; Saxenda Lot #JP54138, both available already in suspension, 6 mg/mL.

## 4. Conclusions

Proper measurement of the native aggregation state of peptide APIs is crucial for their safety and pharmacological profile. This aspect is also confirmed by the requests from the worldwide regulatory agencies to Pharma companies to fully characterize their peptide API and demonstrate sameness with the product in the market (RLD), when the API is a generic. Working in formulation is necessary to appropriately represent samples state. In our work, we addressed the characterization of Liraglutide, a Glucagon-like peptide 1 (GLP1) agonist, by working with an AF4 analytical platform, including UV, fluorescence, refractive index, and multi-angle light scattering detection. The developed method managed to correctly identify Liraglutide aggregation state and provided excellent LoQ and recovery notwithstanding the drawbacks of working with a high-absorbing, high-viscosity eluent. The analysis of a purposely degraded sample confirmed the ability to discern different Liraglutide aggregates in terms of retention and conformation. The developed method was able to identify the association state of Liraglutide in native conditions, which resulted to be pentameric, and was used to perform a sameness study, which confirmed the identity between commercial RLD samples, finished dosed form, and APIs. The developed technology expands the toolkit available for the characterization of the aggregation properties not only of the peptides API, but of protein drugs in general, with the unique advantage of measuring the aggregation behavior in their formulation environment in a native state, since no additional physical medium is used to perform the analysis.
